# What Is Your Diagnosis? Multiple Subcutaneous Lumps in a Horse

**DOI:** 10.1111/vcp.70069

**Published:** 2026-01-16

**Authors:** Federica Meistro, Maria Virginia Ralletti, Riccardo Rinnovati, Alessandro Spadari, Maria Morini

**Affiliations:** ^1^ Department of Veterinary Medical Sciences Alma Mater Studiorum ‐ University of Bologna Bologna Italy

**Keywords:** equine cytology, equine oncology, histiocytic disorders, histiocytic sarcoma

## 1 Case Presentation

A 21‐year‐old Irish Cob gelding was hospitalized, presenting with multiple subcutaneous nodular masses disseminated across the trunk, neck, head, and ventral abdomen. The two larger masses, which presumably appeared about 3 months prior to clinical admission and had been rapidly growing, were located on the lateral aspects of the neck and measured 12 and 10 cm in diameter, respectively (Figure 1). Throughout the rest of the body (head, trunk, withers, both shoulders, and ventral abdomen), the masses exhibited considerable variability in size, ranging from 0.5 to 6 cm in diameter. On dermatologic external examination, all nodules appeared firm, non‐painful, and without pruritus, alopecia, ulceration, or fistulation.

**FIGURE 1:**
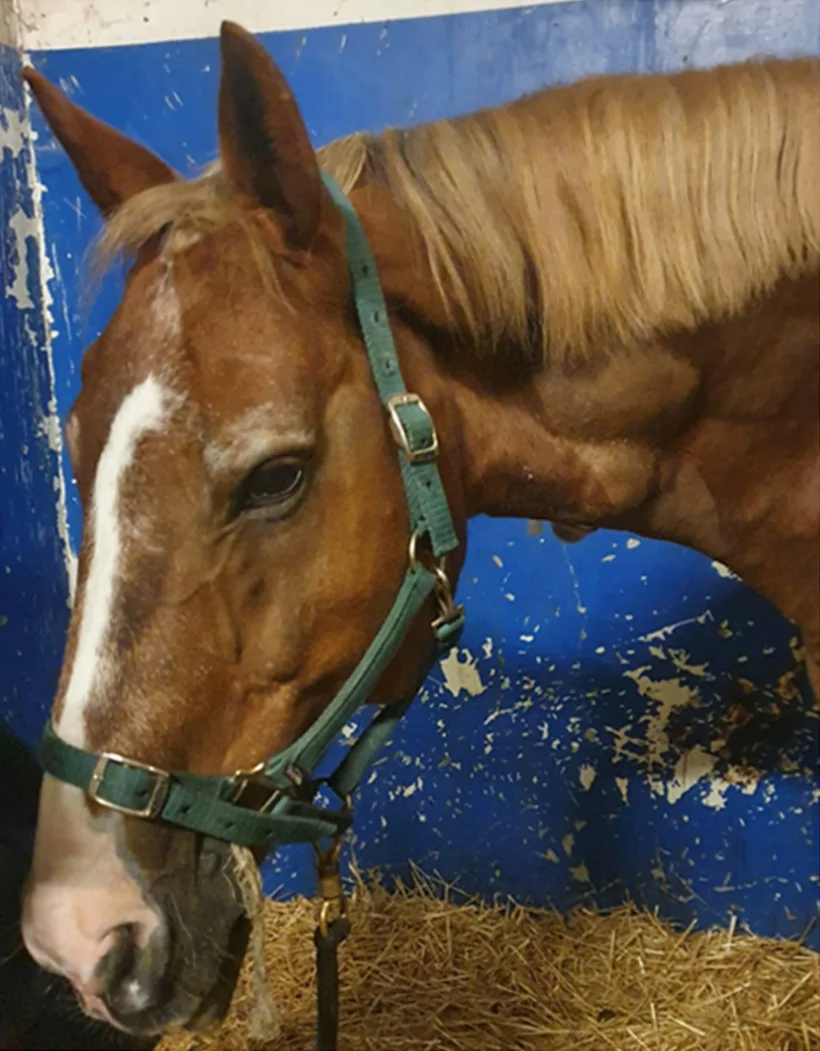
(Left): Macroscopic appearance of masses over the masseter muscle and cervical region.

Fine‐needle aspiration (FNA) cytology was performed on the mass located on the left side of the neck (Figure 2), and concurrently, a surgical excisional biopsy of the same subcutaneous nodule was performed under local anesthesia for histopathologic examination, fixed in 10% neutral buffered formalin, and routinely processed.

**FIGURE 2:**
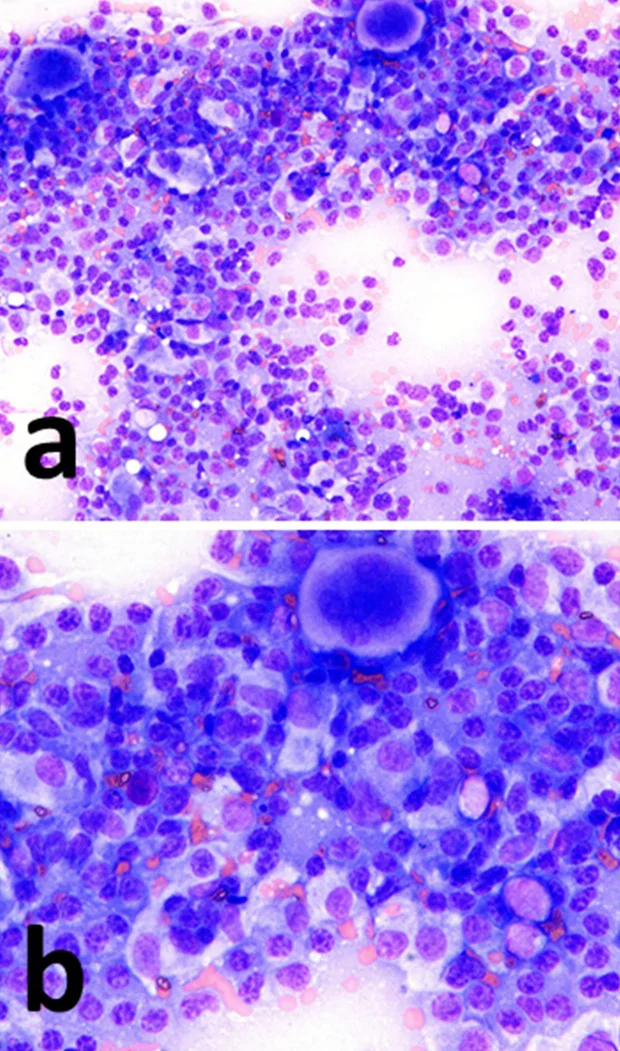
(Right): Fine‐needle aspiration cytology of a subcutaneous neck mass (a: ×10 objective and b: ×40 objective, MGG stain).

### 1.1 Cytologic Interpretation

Malignant Round cell neoplasia with histiocytic differentiation, consistent with histiocytic sarcoma.

The smears were highly cellular, composed of a population of pleomorphic round cells with well‐defined boundaries. The cytoplasm ranged from hyperchromatic to lightly basophilic, typically finely granular with occasional small, optically clear vacuolations. At higher magnification, these cells exhibited a high nucleus‐to‐cytoplasm ratio, well‐defined borders, and variably basophilic cytoplasm. The nuclei varied in size but were predominantly round and centrally located, with reticular chromatin and prominent, often multiple, small round nucleoli. Moderate anisocytosis and anisokaryosis were noted, along with several mitotic figures. A moderate number of binucleate and occasional multinucleate cells (3–8 nuclei, some exhibiting atypia) were also present (Figure 3). The background contained a mild inflammatory infiltrate composed of a moderate number of lymphocytes and plasma cells.

**FIGURE 3:**
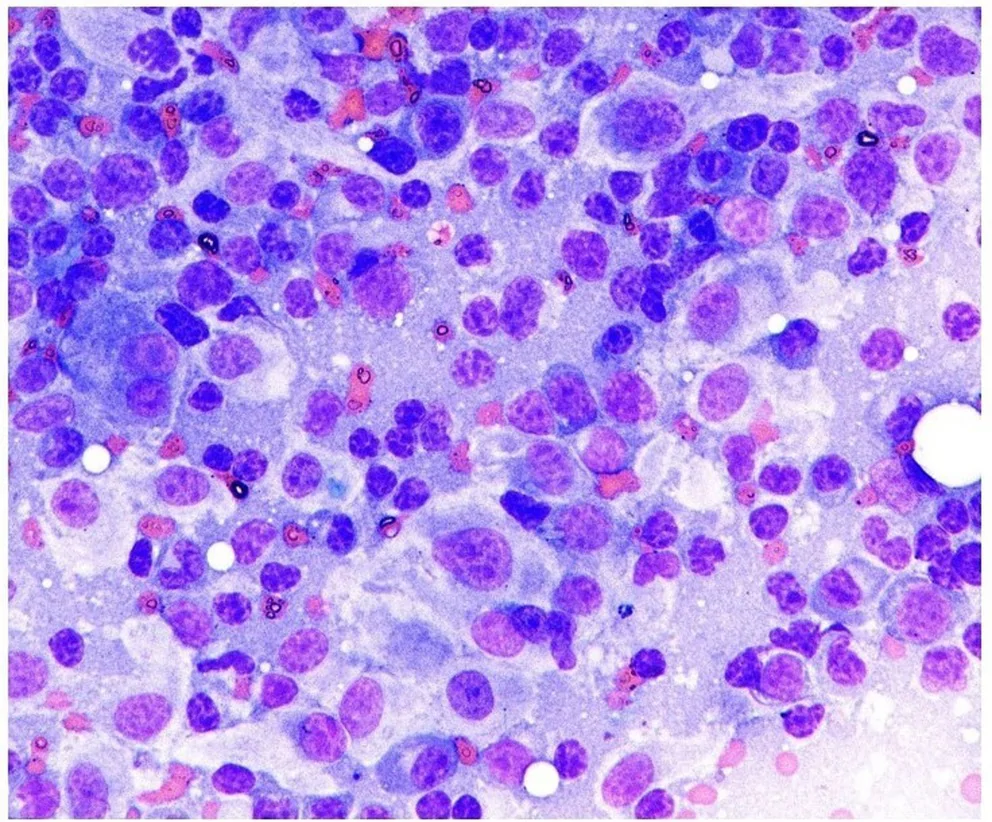
Fine‐needle aspiration cytology of a subcutaneous neck mass (MGG stain). Smears were composed of small clusters and dispersed, discrete, round cells. We also observe large cells (macrocytosis), bi‐ or trinucleated cells, and occasional multinucleated giant cells with 3–5 nuclei (×40 objective).

Based on these cytologic findings, a presumptive diagnosis of a malignant round cell tumor with histiocytic differentiation, consistent with histiocytic sarcoma, was made. Differential diagnoses included equine giant cell tumor of soft part (GCTSP) and histio‐lymphocytic lymphoma.

## 2 Additional Results

Gross examination of the surgical specimen revealed a well‐defined, firm, nodular mass with a gray‐white cut surface. Histopathology disclosed an infiltrative, unencapsulated, highly cellular nodular mass, composed of solid sheets of polygonal to roundish cells supported by scant fibrovascular stroma. The neoplastic cells exhibited spindle to round morphology, measuring approximately 25–30 μm in diameter, with moderately distinct cell borders, an intermediate nucleus‐to‐cytoplasm ratio, and a moderate amount of granular or rarely vacuolated eosinophilic cytoplasm. The nuclei were oval to indented, paracentrally located, and characterized by coarsely granular chromatin and one or two prominent nucleoli. Mitoses were observed at a rate of 23 in 10 HPF (2.37 mm^2^), with moderate anisocytosis and anisokaryosis. A significant number of bi‐ or multinucleated neoplastic cells were present, along with infiltrates of small lymphocytes and plasma cells and multifocal areas of coagulative necrosis (Figure 4a,b).

**FIGURE 4:**
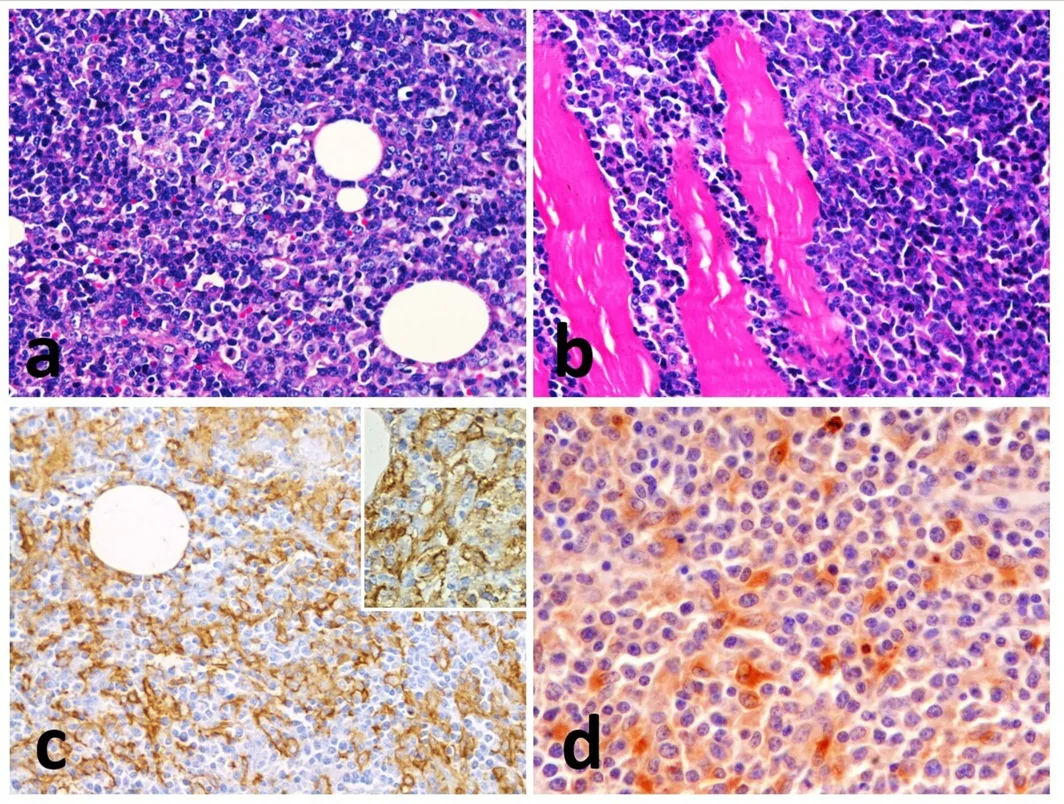
Histology and immunohistochemistry features of the subcutaneous histiocytic sarcoma. (a) Diffuse infiltrate of round to epithelioid, pleomorphic, and histiocytoid tumor cells in an inflammatory background (H&E, ×20 objective). (b) The neoplasm infiltrates and disrupts the subcutaneous muscle bundles (H&E, ×20 objective). Tumor cells showing (c) multifocal moderate to intense cytoplasmic immunoreactivity for IBA‐1 (×20 objective). Inset: At higher magnification (×40 objective) and (d) diffuse and intense cytoplasmic immunoreactivity for lysozyme (×40 objective).

The final diagnosis of histiocytic sarcoma was confirmed via immunohistochemic analysis, which showed strong immunopositivity of the tumor cells for IBA‐1 and lysozyme, in addition to vimentin (Figure 4c,d). Conversely, the neoplastic cells were immunonegative for pan‐cytokeratin, CD3, CD20, and desmin.

## 3 Discussion

The cytologic and histopathologic features observed in this case prompted consideration of three main differential diagnoses: histiocytic sarcoma (HS), cutaneous histiocytic lymphoma, and giant cell tumor of soft part (GCTSP). These entities may share some cytologic or histopathologic features, but differ in cellular composition, behavior, and immunophenotypic profile. However, each of these neoplasms displays distinctive features in terms of cell morphology, tissue architecture, and immunohistochemical profile that support a more precise classification. Histiocytic sarcoma (HS) is a malignant histiocytic proliferative disorder that can present as either a localized or disseminated form. It is predominantly observed in dogs, where it is associated with rapid metastasis and a poor prognosis. HS originates from histiocytic cells, which are specialized leukocytes integral to immune system function. Under various cytokine influences, these cells differentiate into either monocyte/macrophage or dendritic cell lineages [1]. In horses, histiocytic proliferative diseases are rare. Among all documented forms, only the malignant variant has been reported in equine cases [2, 3]. Hematolymphoid neoplasms in horses are uncommon, with lymphoproliferative disorders being more frequent than myeloproliferative disorders. Among them, lymphoma is the most common. To date, only a limited number of cases of equine HS have been documented [2, 4].

In our case, the cutaneous localization appeared to be primary, and no lymphadenopathy was detected on physical examination. However, systemic dissemination could not be definitively excluded, particularly in light of the rapid clinical deterioration observed, which led to euthanasia 1 week after admission. A definitive diagnosis of systemic involvement was not possible, as the owner declined postmortem examination.

A previous report suggests that subcutaneous HS in horses is likely primary in nature [2], exhibiting similar gross and microscopic features to our case. The masses typically appeared encapsulated, firm, roundish, gray‐white, or pinkish in color. Histopathologically, they display marked pleomorphism, with large mononuclear and multinucleated round or polygonal cells [1].

In our case, FNA cytology already pointed toward a diagnosis of histiocytic neoplastic malignancy, confirmed subsequently through histologic and immunohistochemical analyses.

The cytologic characteristics closely resembled those described in canine HS, supporting a diagnosis of a malignant histiocytic tumor, that was additionally supported by the finding of bi‐ and multinucleated elements, intermixed with round histiocyte‐like cells with characteristics of atypia. Among the differential diagnoses initially considered based on the cytologic features, particular attention was given to cutaneous histiocytic lymphoma. This neoplasm, unlike epitheliotropic lymphoma, which is a cutaneous form of lymphoma in horses, is histologically characterized by regions of densely packed lymphocytes interspersed with histiocytes and multinucleated giant cells resembling Langhans cells [1]. In the present case, however, the cytologic smears did not reveal significant lymphocytic infiltration, and the multinucleated cells lacked the characteristic morphology of Langhans giant cells, making the diagnosis unlikely.

Giant cell tumor of soft part (GCTSP) was also considered due to the presence of multinucleated elements in the cytologic preparations. Equine GCTSP is a rare neoplasm primarily affecting older horses, typically manifesting as small, superficial nodules within the hindlimb musculature [5]. Current theories suggest that GCTSP may represent a variant of HS [5], but its low metastatic rate and distinct clinical presentation, associated with the fact that the histiocytic origin is only supposed to be in the multinucleated giant cells, make this hypothesis uncertain. On cytology, GCTSP is characterized by giant multinucleated cells intermixed with spindle‐shaped mesenchymal cells [6]. The cytologic preparations lacked the bizarre multinucleated giant cells typically containing up to 50 nuclei, as well as the prominent population of spindle‐shaped mesenchymal cells characteristic of this tumor. Furthermore, the histopathologic findings of the lesion did not support a diagnosis of GCTSP.

In the equine reports of HS documented by immunohistochemistry [2, 4], the tumor cells exhibit moderate immunopositivity for lysozyme, while showing negativity for other markers, such as CD3, CD20, and desmin [1, 4], thereby confirming the histiocytic origin of the neoplastic cells, as seen in our case. Furthermore, in the case described here the histiocytic origin of the cells is also supported by the immunopositivity to IBA1. Differently, based on their immunophenotype, the neoplastic lymphocytes in lymphoma showed diffuse immunostaining for markers typically expressed by T (CD3) or B (CD20) cells while immunohistochemical evaluation of the GCSTP turned out positive only for vimentin, although the giant cells also tested positive for CD18, supporting the histiocytic origin [5, 6].

It is noteworthy that histiocytic disorders often present clinically similarly to lymphoid tumors [2]. In the present case, fever, weight loss, and extensively spread nodules were the predominant clinical manifestations. Blood analyses in horses diagnosed with HS may reveal pancytopenia [1, 2]; however, this was absent in the gelding under examination.

Equine lymphoid neoplasms or HS almost always have a poor prognosis, and death can occur a few months after diagnosis. Therapy is purely symptomatic. A temporary improvement in cases of neoplasia of other origin can be achieved by administering corticosteroids.

In conclusion, malignant histiocytic neoplastic disorders in horses are rare, but should be considered in cases presenting with multiple subcutaneous nodules. Cytology is a valuable, rapid diagnostic tool to distinguish HS from other cutaneous tumors containing giantlike cells, such as histiocytic lymphoma and GCTSP. Given its aggressive nature and poor prognosis, histiocytic sarcoma must be included in the differential diagnosis when multiple skin masses are observed in horses.

## Conflicts of Interest

The authors declare no conflicts of interest.

## References

[1] D. C. Knottenbelt , J. C. PattersonKane , and K. L. Snalune , Clinical Equine Oncology (Elsevier, 2015).

[2] G. D. Lester , A. R. Alleman , R. E. Raskin , and M. B. Calderwood‐Mays , “Malignant Histiocytosis in an Arabian Filly,” Equine Veterinary Journal 30, no. 5 (1993): 471–473. 10.1111/j.2042-3306.1993.tb02994.x 8223384

[3] C. J. Savage , “Lymphoproliferative and Myeloproliferative Disorders,” Veterinary Clinics of North America. Equine Practice 14, no. 3 (1998): 563–578. 10.1016/s0749-0739(17)30187-6 9891725

[4] O. Paciello , G. Passantino , A. Costagliola , S. Papparella , and A. Perillo , “Histiocytic Sarcoma of the Nasal Cavity in a Horse,” Research in Veterinary Science 94, no. 3 (2013): 648–650, 10.1016/j.rvsc.2013.01.005. 23395306

[5] J. M. Bush and B. E. Powers , “Equine Giant Cell Tumor of Soft Parts: A Series of 21 Cases (2000–2007),” Journal of Veterinary Diagnostic Investigation 20, no. 4 (2008): 513–516, 10.1177/104063870802000418. 18599861

[6] F. Cian , S. Whiteoak , and J. Stewart , “A Case of Giant Cell Tumor of Soft Parts in a Horse,” Veterinary Clinical Pathology 45, no. 3 (2016): 501–504, 10.1111/vcp.12377. 27391215

